# Early Loss of Vision Results in Extensive Reorganization of Plasticity-Related Receptors and Alterations in Hippocampal Function That Extend Through Adulthood

**DOI:** 10.1093/cercor/bhy297

**Published:** 2018-12-07

**Authors:** Mirko Feldmann, Daniela Beckmann, Ulf T Eysel, Denise Manahan-Vaughan

**Affiliations:** 1 Department of Neurophysiology, Medical Faculty, Ruhr University Bochum, Bochum, Germany; 2 International Graduate School of Neuroscience, Ruhr University Bochum, Bochum, Germany

**Keywords:** cortex, GABA, hippocampus, metabotropic glutamate receptor, NMDA receptor, plasticity, sensory loss

## Abstract

Although by adulthood cortical structures and their capacity for processing sensory information have become established and stabilized, under conditions of cortical injury, or sensory deprivation, rapid reorganization occurs. Little is known as to the impact of this kind of adaptation on cellular processes related to memory encoding. However, imaging studies in humans suggest that following loss or impairment of a sensory modality, not only cortical but also subcortical structures begin to reorganize. It is likely that these processes are supported by neurotransmitter receptors that enable synaptic and cortical plasticity. Here, we explored to what extent the expression of plasticity-related proteins (GABA-A, GABA-B, GluN1, GluN2A, GluN2B) is altered following early vision loss, and whether this impacts on hippocampal function. We observed that in the period of 2–4 months postnatally in CBA/J-mice that experience hereditary postnatal retinal degeneration, systematic changes of GABA-receptor and NMDA-receptor subunit expression occurred that emerged first in the hippocampus and developed later in the cortex, compared to control mice that had normal vision. Changes were accompanied by significant impairments in hippocampal long-term potentiation and hippocampus-dependent learning. These data indicate that during cortical adaptation to early loss of vision, hippocampal information processing is compromised, and this status impacts on the acquisition of spatial representations.

## Introduction

Visual sensory loss results in cortical adaptation that manifests itself through changes in neuronal excitability thresholds in the primary sensory cortex affected (Eysel et al. 1999; Quinlan et al. 1999; Giannikopoulos and Eysel 2006; Goel and Lee 2007) and is accompanied by reorganization of neighboring sensory cortices (Goel et al. 2006; Hu et al. 2011; Petrus et al. 2014, 2015). The functional consequences of these adaptations are striking: human subjects that experienced early blindness show superior performance in a multitude of sensory tasks, including tactile acuity, auditory spatial location, spatial navigation, and odor identification (Röder et al. 1999; Van Boven et al. 2000; Goldreich and Kanics 2003; Fortin et al. 2008; Cuevas et al. 2009). These changes are supported by processes that underlie cortical plasticity (Bear et al. 1986). In the somatosensory cortex, for example, expanded cortical representations of the “reading” finger have been identified in Braille readers (Pascual-Leone and Torres 1993). A stable enlargement of the sensorimotor representation of the index finger, which enables fluent reading, develops over the course of at least 10 months however (Pascual-Leone et al. 1999), suggesting that sensory enhancements that develop after blindness involve gradual and cumulative adaptations. In line with this assumption, anatomical studies revealed an expansion of barrel fields in mice 2–3 months after binocular enucleation (Rauschecker et al. 1992). Early visual deprivation of cats leads to plasticity in the anterior ectosylvian cortex, and visual cortical neurons of animals with an intact visual system respond to other sensory properties, suggesting that they engage in adaptive plasticity (Rauschecker and Korte 1993). Moreover, functional magnetic resonance imaging studies revealed that sensory loss leads to the gradual inclusion of additional sensory structures in tasks that they would not normally process, such as the recruitment of the occipital cortex for Braille reading (Sadato et al. 1996; Cohen et al. 1999; Merabet et al. 2009), or sound localization in adults blinded in early life (Kujala et al. 1995; Weeks et al. 2000). Many studies have addressed how the visual cortex responds to visual deprivation during the critical period (Espinosa and Stryker 2012; Takesian and Hensch 2013) and it has been proposed that injury to the visual cortex results in a reactivation of critical period mechanisms related to enhanced cortical plasticity (Levelt and Hübener 2012; Nahmani and Turrigiano 2014). Thus, a wealth of the literature describes early developmental changes in the visual cortex related to the critical period and/or visual deprivation during this phase, as well as long-term effects of blindness on sensory acuity and spatial skills. Few studies have addressed how crossmodal neuroplasticity (that is triggered by blindness) correlates with changes on a molecular level. Furthermore, although it is clear that “long-term” adaptation supports marked improvements in sensory acuity in intact modalities, in conjunction with improvements in sensory and spatial representations, and that visual deprivation during the critical period impacts on cortical plasticity, little is known about the intermediate consequences, for the “adult” brain, of sensory loss on structures that are pivotal to the generation of complex sensory and spatial representations.

Blind individuals often exhibit exceptional spatial navigation skills (Fortin et al. 2008). This is largely the domain of the hippocampus, a structure that uses sensory information to generate spatial representations by means of place fields (Save et al. 2000; Zhang and Manahan-Vaughan 2015) and synaptic plasticity (Kemp and Manahan-Vaughan 2004, 2008, 2012; André and Manahan-Vaughan 2013; Dietz and Manahan-Vaughan 2017). Interestingly, magnetic resonance imaging studies have revealed a larger hippocampal volume in blind individuals, as a result of increased use of memory function during spatial learning tasks (Fortin et al. 2008). Adaptation to blindness is a gradual process however, requiring not only that the individual adjusts to the absence of the visual input but also that the hippocampus learns to rely on nonvisual sensory modalities as the primary source of spatial reference input. As yet, it is not clear to what extent the hippocampus engages in reorganization following the onset and perpetuation of blindness, but studies in rodents have shown that in the absence of reliable visual information, the hippocampus readily uses available sensory modalities for both spatial navigation (Zhang et al. 2014; Draht et al. 2017) and for the encoding of spatial memory (André and Manahan-Vaughan 2013; Dietz and Manahan-Vaughan 2017).

Spatial information processing is not the sole domain of the hippocampus: it is also enabled by association cortices, such as the posterior parietal cortex. This structure supports the building of spatial representations by integrating sensory information of different sensory modalities (Andersen 1997) and the guidance of movement in space (Pinto-Hamuy et al. 2004). It also may function as a binding element between the somatosensory and occipital cortices when the congenitally blind perform tactile perception tasks (Leo et al. 2012). Considering that the hippocampus and the posterior parietal cortex rely heavily upon visual input in sighted individuals, it is likely that neural plasticity occurs also in these 2 structures following blindness. We hypothesized that these processes are supported by neurotransmitter receptors that are critical for the expression of synaptic and cortical plasticity. Receptors that are particularly plasticity-relevant, comprise the N-methyl-D-aspartate receptors (NMDAR) (Nicoll and Malenka 1999; Collingridge et al. 2004) and the gamma-aminobutyric acid (GABA) receptors (Collingridge et al. 2004). Whereas NMDAR are essential for hippocampal synaptic plasticity (Bliss and Collingridge 1993), GABA receptors have been proposed to support rewiring between cortical modalities and thereby, crossmodal plasticity (Desgent and Ptito 2012).

Several studies have addressed the short-term consequences of visual deprivation on e.g., glutamatergic transmission in the primary visual cortex (Eysel et al. 1999; Arckens et al. 2000) and have reported that experience-dependent shifts in the thresholds for long-term potentiation (LTP) and long-term depression are triggered in the cortex that are supported in turn by regulation of NMDAR subunit composition (Kirkwood et al. 1996; Quinlan et al. 1999; Yashiro and Philpot 2009). Little is known, however, about the long-term impact of visual deprivation on plasticity-relevant receptors. Furthermore, a possible reorganization of plasticity-relevant receptors in sensory cortices that are not the main site of processing of the absent modality, in integrative structures such as the posterior parietal cortex, or cognitive structures such as the hippocampus, has not been examined in detail.

The first aim of this study was to examine whether progressive alterations in plasticity-related neurotransmitter receptors occur in early adulthood in rodents that develop postnatal blindness. Knowledge in this regard will help us understand both the initial mechanisms underlying cortical adaptation to blindness as well as the initial adaptive steps instigated within the hippocampus. We scrutinized multiple sensory cortices, the posterior parietal cortex, and the hippocampus in a mouse strain that experiences total visual loss (due to a congenital retinal degeneration 1 (rd1) mutation) by approximately 4 weeks postnatally. We observed that the GluN2B subunit of the NMDAR was the most profoundly affected, with increases in expression becoming manifest as early as 2 months postnatally in localized regions of the hippocampus, followed by increases in expression in all sensory cortices, hippocampal areas, and the posterior parietal cortex occurring by 4 months. More localized decreases in the expression of GABA receptor subunits also occurred. The second aim of the study was to examine to what extent early blindness affects hippocampal information processing. Strikingly, receptor changes triggered by early blindness were accompanied by a significant impairment of hippocampal LTP and of object recognition learning in early adulthood.

Taken together our data suggest that an extensive reorganization of plasticity-relevant neurotransmitter receptors occurs following early postnatal blindness that progresses through young adulthood. Reorganization of the expression of NMDAR and GABA receptors in the sensory cortices is likely to reflect their restructuring in terms of a functional reassignment of sensory processing tasks during adaptation to blindness. The finding that receptor expression changes in the hippocampus precede changes in the cortex may reflect the massive and rapid adaptation in spatial representations and spatial information processing that will inevitably occur following the manifestation of blindness. Strikingly, the hippocampus is initially overwhelmed by this process. The impairments of LTP we observed may reflect changes in induction thresholds or the frequency dependency of synaptic plasticity that underlies adaptive encoding of space in the absence of visual inputs. This suggests in turn that the adaptation of the hippocampus to the absence of the visual modality, and the ultimate improvements of spatial perception detected in blind individuals, is a long-term, experience-dependent, and gradual process.

## Materials and Methods

### Animals

The present study was carried out in accordance with the European Communities Council Directive of 22 September 2010 (2010/63/EEC) for care of laboratory animals. Male CBA/J (Charles River) and CBA/CaOlaHsd mice (Harlan (Envigo) Laboratories) were group housed in a temperature- and humidity-controled vivarium with a constant 12-h light–dark cycle (lights on from 6 AM to 6 PM) with ad libitum food and water access. The CBA/J (PDE6brd1/PDE6brd1) mouse expresses a hereditary mutation of the *PDE6B *gene (rd1) (Pittler and Baehr 1991; Cohen et al. 2001). The CBA/CaOlaHsd mouse (in contrast, for example, to the C57BL/6 mouse) has a circadian cycle-sensitive visual system (Jin and Ribelayga 2016), with no reported deficits in other sensory modalities (Brooks et al. 2004; Walton et al. 2008; Heckman et al. 2017) and equivalent cognitive abilities to a variety of lab mouse strains (Brooks et al. 2005).

### Tissue Preparation

Mice were euthanized at the age of 2 and 4 months using isoflurane for inhalational anesthesia, followed by an intraperitoneal injection with sodium pentobarbital. Transcardial perfusion was conducted with cooled, 0.2% heparinized Ringer solution for 10 min, followed by 4% paraformaldehyde (PFA) in phosphate-buffered saline (PBS) for 10 min. Brains were collected and immersed in PFA solution for 24 h at 4°C, then in 30% sucrose in PBS for storage. Frozen sections of 30 μm were prepared with a cryostat microtome (Leica) for Nissl staining with cresyl violet, and immunohistochemistry. Slices from CBA/J and CBA/CaOlaHsd mice were processed together to minimize interarray variations between different staining sets. Nissl stainings were prepared to check tissue quality and to choose slices of the correct bregma distance, as well as to examine the extent of retinal degeneration or viability.

### Immunohistochemistry

Three different protocols were used for immunohistochemistry depending on the primary antibody: GABA-A, GABA-B, and GluN2B immunostainings were performed with an avidin–biotin complex (ABC) method as described by Heras et al. (1995). GluN1 subunit staining was performed with tyramine amplification, in addition to the ABC method, as described by Adams (1992). For GluN2A subunit assessment, we used a protocol that implemented streptavidin enhancement. Negative controls comprising tissue incubations with separate primary and secondary antibodies were performed in order to verify that specific binding had occurred.

For immunohistochemistry using the ABC method according to Heras et al. (1995), free floating sections were rinsed in a dilution medium composed of PBS thrice for 10 min. Sections were thus washed after each step of the protocol. Next, sections were placed in 0.3% H_2_O_2_ for 20 min to remove endogenous peroxidase activity, thereby ensuring that background staining could be kept to a minimum. Afterward, sections were preincubated with blocking solution containing 20% avidin (avidin–biotin blocking kit, Vector Laboratories), 10% normal serum (Vector Laboratories), and 0.2% Triton X-100 (Tx) for 90 min to reduce nonspecific binding. Sections were then incubated overnight at room temperature with the primary antibody solution, containing 20% biotin (avidin–biotin blocking kit, Vector Laboratories), 1% normal serum, 0.2% Tx, and the relevant primary antibody: GABA-A (1:400, monoclonal mouse antibody (AB), MAB341, Merck Millipore), GABA-B (1:500, monoclonal mouse AB, ab55051, Abcam), or GluN2B (polyclonal goat AB, sc-1469, Santa Cruz Biotechnology). The secondary antibody was applied for 90 min. We used a biotinylated horse–antimouse antibody for GABA-A and GABA-B (1:500, BA-2001, Vector Laboratories) and a biotinylated horse–antigoat antibody for GluN2B (1:500, BA-9500, Vector Laboratories). Sections were then immersed in 1:1000 ABC-Elite detection system (Vector Laboratories), 1% normal serum, and 0.1% Tx for 90 min. Finally, the staining reaction was performed with 3,3′-diaminobenzidin (DAB, Sigma-Aldrich) in 0.01% hydrogen peroxide PBS for 10 min.

For immunohistochemistry using the ABC method including tyramine amplification, according to Adams (1992), Tris (hydroxymethyl)-aminomethan-buffered saline (TBS) was used instead of PBS, and bovine serum albumin (BSA, Sigma-Aldrich) was used instead of n-serum. The initial steps of the protocol were analogous to those described above for GABA and GluN2B assessment. Primary antibody incubation specific to GluN1 (monoclonal rabbit AB, 1:200, AB9864R, Merck Millipore) was extended to 5 days at 4°C. A biotinylated goat–antirabbit antibody (1:500, BA-1000, Vector Laboratories) was used as secondary antibody. Tyramine amplification was performed as described by Adams (1992) and followed by DAB staining.

GluN2A subunits were detected using streptavidin enhancement. TBS and normal serum were used as dilution medium. The initial steps were the same as those described above. Incubation with the primary antibody specific to GluN2A (1:250, polyclonal rabbit AB, sc-9056, Santa Cruz Biotechnology) lasted for 24 h at room temperature, followed by secondary antibody incubation with biotinylated goat–antirabbit antibody (1:500, BA-1000, Vector Laboratories). Afterward, sections were incubated with 1:1000 streptavidin (Cy™3-conjugated streptavidin, Jackson Laboratories) for 30 min. Antistreptavidin antibody (1:500, biotinylated goat–antistreptavidin, BA-0500, Vector Laboratories) was added for another 30 min, followed by DAB staining.

### Behavioral Experiments

Behavioral experiments were conducted with 16–20-week-old CBA/J and CBA/CaOlaHsd mice.

On the day before object recognition training commenced (Fig. 5*A*), mice were placed inside the test arena for a habituation phase of 10 min. Procedures followed those that were previously reported (Goh and Manahan-Vaughan 2013): during the training trial, mice were presented with 2 identical novel objects for 10 min. Five minutes, 1 h, and 24 h later, one of the familiar objects was exchanged for a novel object in the same position. Each test trial lasted 10 min, during which both the total exploration time, as well as the relative exploration time of the familiar and the novel objects were measured. Objects and test arenas were cleaned thoroughly in the intervals between testing to remove any odor traces. Multiple identical examples of each object were available. The experiments were recorded using a camera that was positioned above the test arena to enable subsequent, off-line, experimenter-blind analysis.

To examine to what extent the CBA/CaOlaHsd mice might benefit from visual stimuli, we compared their behavior in both fully illuminated (400 lux) and dark conditions (10 lux). To enable camera recordings, illumination was provided by long wavelength red light (>700 nm). Mice are myopic (Geng et al. 2011) and 97% of their photoreceptors are rods (Carter-Dawson and LaVail 1979; Jeon et al. 1998). Mouse cones are composed of 2 distinct populations (Rohlich et al. 1994; Haverkamp et al. 2005; Nikonov et al. 2006): genuine S-cones that express only S-opsin and have a peak sensitivity to UV light (360 nm) (Jacobs et al. 1991), and coexpressing cones, that in addition to S-opsin, express M-opsin that exhibits peak sensitivity to green light (508 nm) (Wang et al. 2011) but can also weakly detect light up to wavelengths of approximately 650 nm (Jacobs et al. 2004). Mice cannot however perceive long-wave red light as it is out of the range of their spectral vision (Wang et al. 2011).

### Electrophysiology

Two- or 4-month-old mice were deeply anesthetized with isoflurane and then decapitated. Brains were dissected in cold (4°C), oxygenated artificial cerebrospinal fluid (aCSF) using 87 mM NaCl, 2.4 mM KCl, 1.3 mM MgSO_4_, 0.5 mM CaCl_2_, 26 mM NaHCO_3_, 1.25 mM NaH_2_PO_4_, and 2 mM d-glucose. Following brain dissection, slices (400 μm) were cut using a vibratome and stored on a nylon net in a holding chamber filled with aCSF and glucose (30°C) for 30 min. Afterward, the slices were transferred to an interface recording chamber (Scientific Systems Design Inc). This system was continuously perfused with oxygenated aCSF (95% O_2_, 5% CO_2_, 125 mM NaCl, 3 mM KCl, 2.5 mM CaCl_2_, 1.3 mM MgSO_4_, 1.25 mM NaH_2_PO_4_, 13 mM d-glucose, 26 mM NaHCO_3_) at the rate of 1.5 mL/min. The temperature in the chambers was kept at 30 ± 2°C by an automatic temperature controller (PTC03, Scientific Systems Design Inc). The slices were allowed to acclimatize for 1 h before recordings started. A bipolar stimulation electrode (Fredrick Haer) was positioned in Stratum radiatum of CA1 and a borosilicate glass-micropipette (1–2 MΩhm) filled with aCSF served as a recording electrode that was placed in the CA1 dendritic area. Biphasic test pulse stimuli (0.025 Hz, duration: 0.2 ms) were applied and for each time point 5 evoked responses were averaged. The sample rate for the recording of the evoked signals was set at 16 000 Hz. An input/output curve (I/O curve), using a stimulation range from 60 to 600 μA was conducted, and for experiments a stimulation intensity that evoked 50% of the I/O maximum was used. Basal synaptic transmission (“baseline”) was recorded for 60 min and then, without changing the stimulus intensity, LTP was induced via high-frequency stimulation (HFS, 3 trains of 100 pulses at 100 Hz, delivered 5 min apart). Evoked responses were followed for 2 h after HFS.

### Retinal Assessments

**Figure 1.**
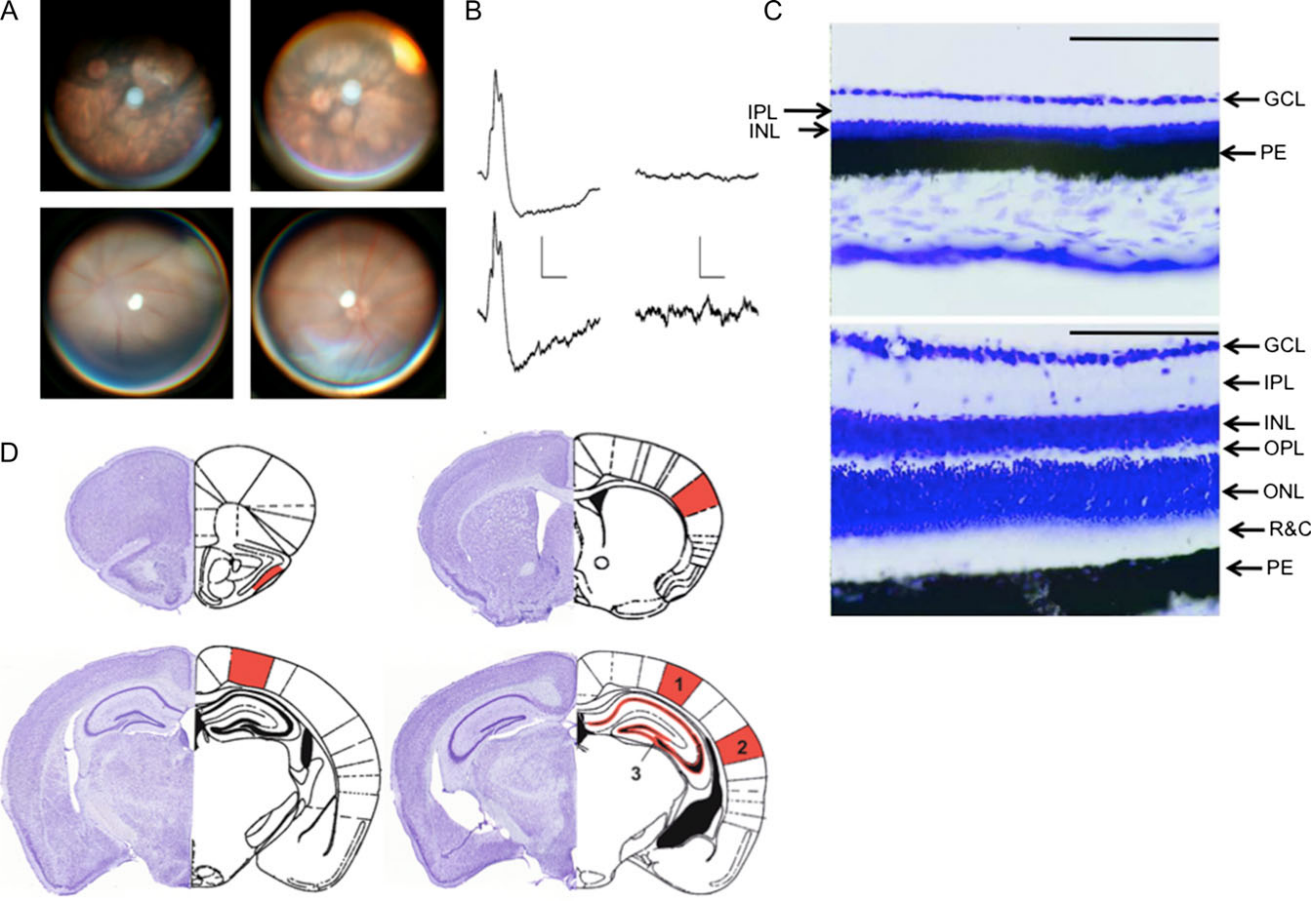
Retinal viability of CBA/J and CBA/CaOlaHsd mice and schema showing areas of interest. (*A*) Fundus photographs of CBA/J and CBA/CaOlaHsd mice. Top: The retina of CBA/J mice exhibits typical signs of the rd1 mutation. The fundus of the eye appears map-like with large patches of depigmentation. Retinal vessels are clearly narrowed as a sign of vascular sclerosis. At the age of 4–5 weeks (top left) retinal degeneration is already complete. Top right is an example of the fundus of a 7–8-week-old mouse. Bottom: Fundus photographs from CBA/CaOlaHsd mice show normally pigmented retina at 4–5 weeks (left) and 7–8 weeks (right) of age. Blood vessels show regular configuration with large retinal venules and smaller retinal arterioles. (*B*) Analog examples of electroretinograms recorded from 4 to 5-week-old CBA/CaOlaHsd (left) and CBA/J mice (right). CBA/J mice failed to exhibit electrophysiological retinal activity. Vertical scale bars: 50 μV, horizontal scale bars: 100 ms. (*C*) Histological assessment of CBA/J and CBA/CaOlaHsd retinae. Top: CBA/J mice retina reveals degeneration of all retinal layers. Bottom: CBA/CaOlaHsd retina display a normal configuration. GCL: ganglion cell layer, IPL: inner plexiform layer, INL: inner nuclear layer, OPL: outer plexiform layer, ONL: outer nuclear layer, R&C: rods & cones, PE: pigment epithelium. Scale bar 100 μm. (*D*) The areas scrutinized in the CBA/J mouse cerebral cortex and hippocampus are shown in the cresyl violet-stained histological sections (left side of each example). The specific regions examined are indicated in the schemas on the right side of exach example (based on Franklin and Paxinos, 2008). Markings in red represent the following areas: Top left: piriform cortex. Top right: somatosensory cortex. Bottom left: posterior parietal cortex. Bottom right: visual cortex (1), auditory cortex (2), and hippocampus (3) including the dentate gyrus, and cornus ammonis subregions (CA1–CA3).

By means of ophthalmoscopy, fundus photography were performed on 4–5- and 7–8-week-old CBA/J and CBA/CaOlaHsd mice. Animals were anesthetized with an intraperitoneal injection of ketamine 100 mg and xylazine 5 mg in saline solution/kg body weight. After the mouse was deeply anesthetized, 2–3 drops of 1 % atropine sulfate solution were applied on each eye to widen the pupils for a better view of the retina. The photographs were taken with a handheld fundus camera (Genesis D). Animals with a fundus indicating retinal degeneration as described by Chang et al. (2002), were considered degeneration-positive and blind in comparison to animals that showed a normal fundus (Fig. 1*A*).

Electroretinography (ERG) was conducted in age-matched CBA/J and CBA/CaOlaHsd mice to assess retinal function (Heckenlively et al. 1989; Dalke et al. 2004). Mice were anesthetized with Ketanest/Xylazine (100 mg ketamine, 5 mg xylazine per kg body weight) and gently placed in a box with light gray walls. For ERG recordings, a silver wire (0.2 mm) was placed paracorneally on one eye. Silver clamps were attached to the ear to serve as an indifferent electrode and to the tail for grounding. Right and left eyes were investigated separately. Background luminance was kept at a low photopic level (5 cd/m^2^, comparable to the values measured in the box for the object recognition task). A light-emitting diode (LED) stroboscope (Photic, LifeLines Neurodiagnostic systems) was placed 0.2 m in front of the eye and used in triggered single flash mode (flash duration 1 ms, luminous power 900 lm). Full field flashes were applied at 1 Hz (10 blocks with 50 stimuli separated by 10 s), responses were amplified (gain 1000×, bandpass filter 1–5000 Hz, differential amplifier, Model 1700, A-M Systems), sampled for poststimulus periods of 500 ms at 5 kHz (CED 1401 Micro 3, Cambridge electronic design; recording software Intracell 1.5, developed at Leibniz Institute for Neurobiology), and averaged over all responses.

For histological assessments, 25-μm retinal cryosections were mounted on Super Frost Plus slides (Thermo Fisher Scientific), dried overnight, and stained with cresyl violet. Sections revealed degeneration of all retinal layers in rd1-positive animals as compared with normal retinal structures in the control animals (Fig. 1*C*).

### Data Analysis

For the analysis of neurotransmitter receptor distribution, we used optical density measurements in the specific areas with an image-based analysis system (Neurolucida, MBF Bioscience). Although DAB-stained tissue does not follow Lambert–Beer’s law, a standardized immunoassay allows protein quantification (Grube 2004) and was conducted here. Cortical and hippocampal fields were assessed at the following distances from bregma: 2.2 mm (Fig. 1*C*, piriform cortex), 0.5 mm (Fig. 1*C*, somatosensory cortex), −1.8 mm (Fig. 1*C*, posterior parietal cortex), −2.5 mm (Fig. 1*C*, auditory cortex, visual cortex, dentate gyrus, CA1–3). All slides were observed under a Leitz Wetzlar Dialux 20 brightfield microscope (Leica) with a 2.5-fold magnification. Whole slide images were acquired with a digital camera (QIC-F-CLR-12, QImaging), using the virtual tissue 2D module of Neurolucida. Immunohistochemical background staining was corrected by subtracting density values obtained in the corpus callosum to minimize interarray differences. Luminance information ranging from 0 to 255 was obtained from the whole area.

For immunohistochemical samples, all data were tested for between-group effects with a multifactorial analysis of variance (ANOVA) followed by a post hoc Duncan’s test. Between-group factors, strain (CBA/J vs. CBA/CaOlaHsd) and brain area (piriform cortex: PiC, somatosensory cortex: SC, posterior parietal cortex: PPC, visual cortex: VC, auditory cortex: AuC, dentate gyrus: DG, CA1, CA2/3, CA4), were assessed 2 and 4 months postnatally.

The recordings of the behavioral experiments were analyzed by measuring old object and novel object exploration times separately. Object exploration was defined as the animals touching or sniffing the object, or when the snout was near the object (<2 cm) and pointed directly toward it. The recognition index was calculated by determining object exploration as a percentage of the total exploration time (Clarke et al. 2010; Goh and Manahan-Vaughan 2013). The behavioral data were analyzed using ANOVA to assess the exploration time of old and novel objects and to compare exploration behavior of CBA/J and CBA/CaOlaHsd mice. This was done by comparing group means with the fixed value of 50%, which represents no differentiation between objects. An ANOVA with repeated measures was used to assess behavior of the animals under illuminated and dark conditions.

For electrophysiological data, a 2-way ANOVA with repeated measures was applied to test for between-group effects. A post hoc Fisher’s test was used to determine which comparisons generated significant differences, if appropriate. The number of animals is signified by an upper case, “N,” the number of slices used is signified by a lower case “n.”

All data were shown as mean ± standard error of mean. All statistical tests were performed using STATISTICA 12 (Statsoft). The level of significance was set at *P* < 0.05.

## Results

### Retinal Assessments Reveal that 4–5-Week-Old CBA/J Mice are Completely Blind

The congenital rd1 mutation that occurs in CBA/J mice causes severe retinal degeneration (Chang et al. 2002). To ascertain the degree of retinal degeneration in young CBA/J mice, we conducted ophthalmological assessments of the retinae of CBA/J (*N* = 8) and CBA/CaOlaHsd mice without retinal degeneration (*N* = 8) at 4–5 weeks and 7–8 weeks postnatally. We observed that at these time points almost complete degeneration of the retina of CBA/J mice was evident (Fig. 1*A*) compared with the retina of control mice that possessed retinae that were completely intact (Fig. 1*A*). Histological assessment of the retinae of 4–5-week-old CBA/J and CBA/CaOlaHsd mice confirmed that retinal degeneration had occurred (Fig. 1*C*): retinal thickness was reduced and many characteristic layers could no longer be identified.

To verify that the retinal degeneration detected using ophthalmological and histological analysis corresponds to the manifestation of early postnatal blindness in CBA/J mice, we conducted ERG recordings to test for electrophysiological responses in CBA/J and CBA/CaOlaHsd mice (Fig. 1*B*). In CBA/CaOlaHsd mice (*N* = 4) a small a-wave was followed by b-waves with amplitudes of 63–83 μV (Fig. 1*B*). In CBA/J mice (*N* = 4), the ERG recordings showed a flat line (Fig. 1*B*) corresponding to complete absence of retinal responses, consistent, in turn, with blindness. These findings are consistent with reports by others that CBA/J (rd1) mice exhibit flat ERG responses as early as 4 weeks postnatally (Roderick et al. 1995).

### GluN2B Subunit Expression is Increased in the Hippocampal CA4 Region of CBA/J Mice at 2 Months of Age. Expression of Other NMDAR Subunits, or GABA Receptors, is Unchanged

To examine to what extent the early manifestation of blindness in CBA/J mice results in cortical and hippocampal organization, we used an immunohistochemical approach to scrutinize neurotransmitter receptor expression in primary and association sensory cortices, as well as in the hippocampus (Fig. 1*D*). We focussed specifically on the expression subunits of the NMDAR, as well as GABA-A and GABA-B receptors.

In 2-month-old CBA/J mice (*N* = 6), GluN2B expression was significantly increased in the hippocampus compared to control (CBA/CaOlaHsd) mice (*N* = 6). This effect was specific to the hippocampal CA4 region (see statistical report in Table 1, and optical density values in Supplementary Table 1). None of the other neurotransmitter receptors or receptor subunits assessed (GluN1, GluN2A, GABA-A, GABA-B) exhibited any changes in expression at this postnatal age (Supplementary Figs S1, S2, Supplementary Table 1).

**Table 1.** Statistical comparison of neurotransmitter receptor expression in CBA/J and CBA/CaOlaHsd mice 2 and 4 months postnatally

Receptor	Area	CBA/J	CBA/CaOlaHsd	*P*	*N*
2 Months					
GluN2B	CA4	28.85 ± 3.61	18.28 ± 2.65	<0.05	6
4 Months					
GluN2B	PiC	30.12 ± 2.07	12.27 ± 1.82	<0.001	5
	SC	39.38 ± 2.39	20.08 ± 1.76	<0.001	5
	PPC	46.93 ± 3.28	26.93 ± 1.94	<0.001	5
	VC	39.51 ± 3.10	23.86 ± 1.35	<0.001	5
	AuC	35.38 ± 3.97	20.03 ± 1.71	<0.001	5
	DG	23.68 ± 2.91	11.97 ± 1.30	<0.01	5
	CA1	37.72 ± 2.29	23.28 ± 1.28	<0.001	5
	CA3	36.61 ± 3.20	19.81 ± 1.80	<0.001	5
	CA4	22.93 ± 3.47	10.25 ± 1.48	<0.01	5
GABA-A	SC	86.89 ± 4.20	104.76 ± 6.06	<0.05	6
	PPC	65.16 ± 7.33	85.80 ± 5.10	<0.05	6
	DG	75.75 ± 4.18	99.29 ± 4.72	<0.01	6
	CA1	64.73 ± 3.13	84.96 ± 5.75	<0.05	6
GABA-B	PiC	32.67 ± 3.95	22.72 ± 1.90	<0.05	5

### By 4 Months of Age, GluN2B Subunit Expression is Increased in All Hippocampal Subregions, as well as in the Sensory Cortices of CBA/J Mice. GluN1 and GluN2A Subunit Expression is Unchanged

**Figure 2.**
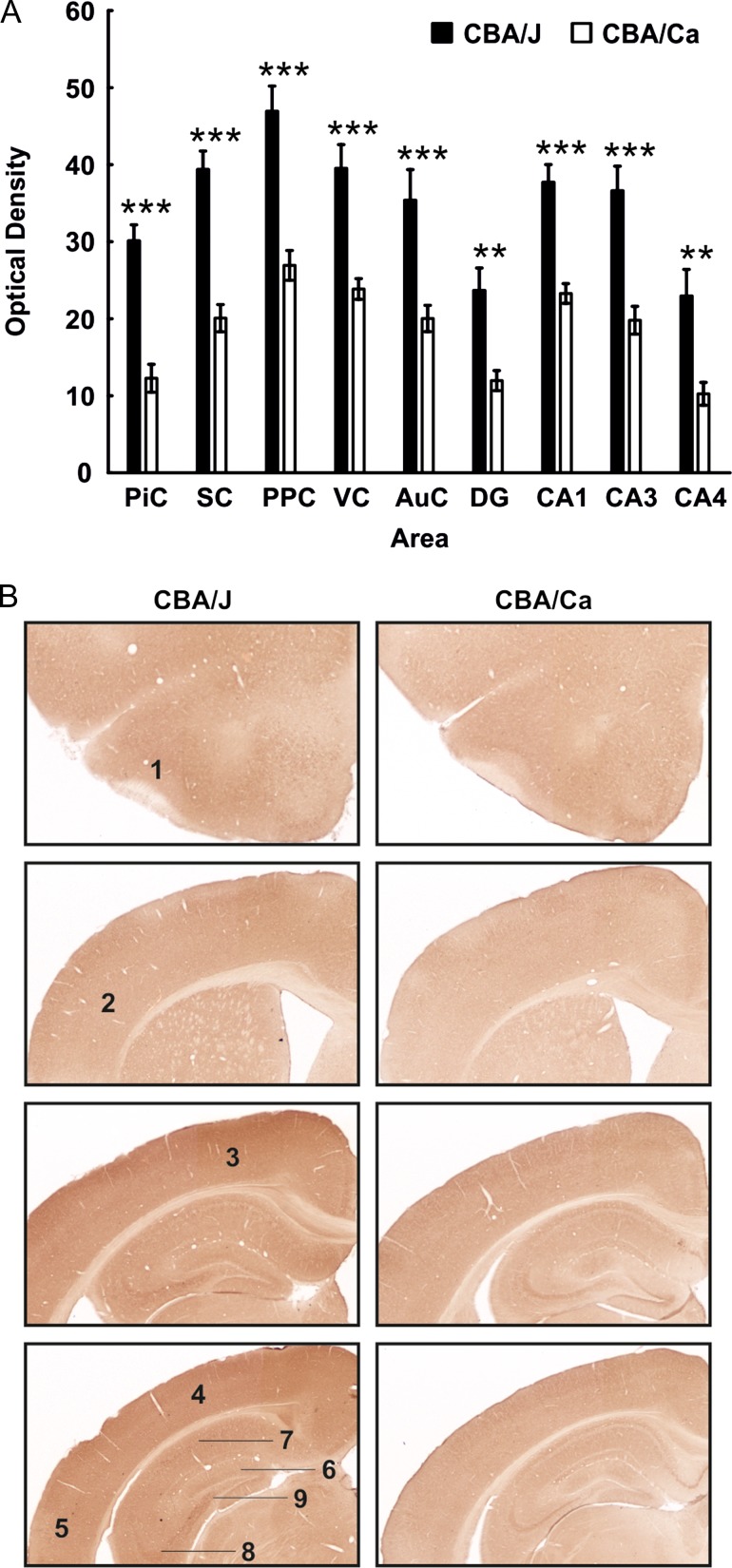
GluN2B expression is globally increased in CBA/J mice 4 months postnatally. (*A*) Bar charts represent mean GluN2B receptor optical densities in different brain regions 4 months postnatally in CBA/J mice compared with (CBA/CaOlaHsd) controls. Receptor density is significantly increased in the piriform cortex (PiC), somatosensory cortex (SC), posterior parietal cortex (PPC), visual cortex (VC), auditory cortex (AuC), dentate gyrus (DG), CA1, CA3, and CA4. Data are means ± SEM. *P < 0.05; **P < 0.01; ***P < 0.001. (*B*) DAB-stained coronary sections showing GluN2B expression in CBA/J and control mice 4 months postnatally. The photomicrographs highlight the changes as described in (*A*). 1: piriform cortex, 2: somatosensory cortex, 3: posterior parietal cortex, 4: visual cortex, 5: auditory cortex, 6: dentate gyrus, 7: CA1, 8: CA3, 9: CA4.

In 4-month-old CBA/J mice, significant increases in GluN2B expression (*N* = 6) became evident in all subfields of the hippocampus (Fig. 2), as well as in all primary sensory cortices and the posterior parietal cortex (Fig. 2) compared with expression levels detected in CBA/CaOlaHsd mice (*N* = 6) (Table 1). GluN1 and GluN2A expression were equivalent in the cortex and hippocampus of CBA/J and control mice at this stage of maturity (Supplementary Table 1).

### By 4 Months of Age GABA-A Expression is Decreased in the Hippocampal CA1 and Dentate Gyrus Subregions, as well as in the Somatosensory and Posterior Parietal Cortices of CBA/J Mice. GABA-B Expression is Increased in the Piriform Cortex

**Figure 3.**
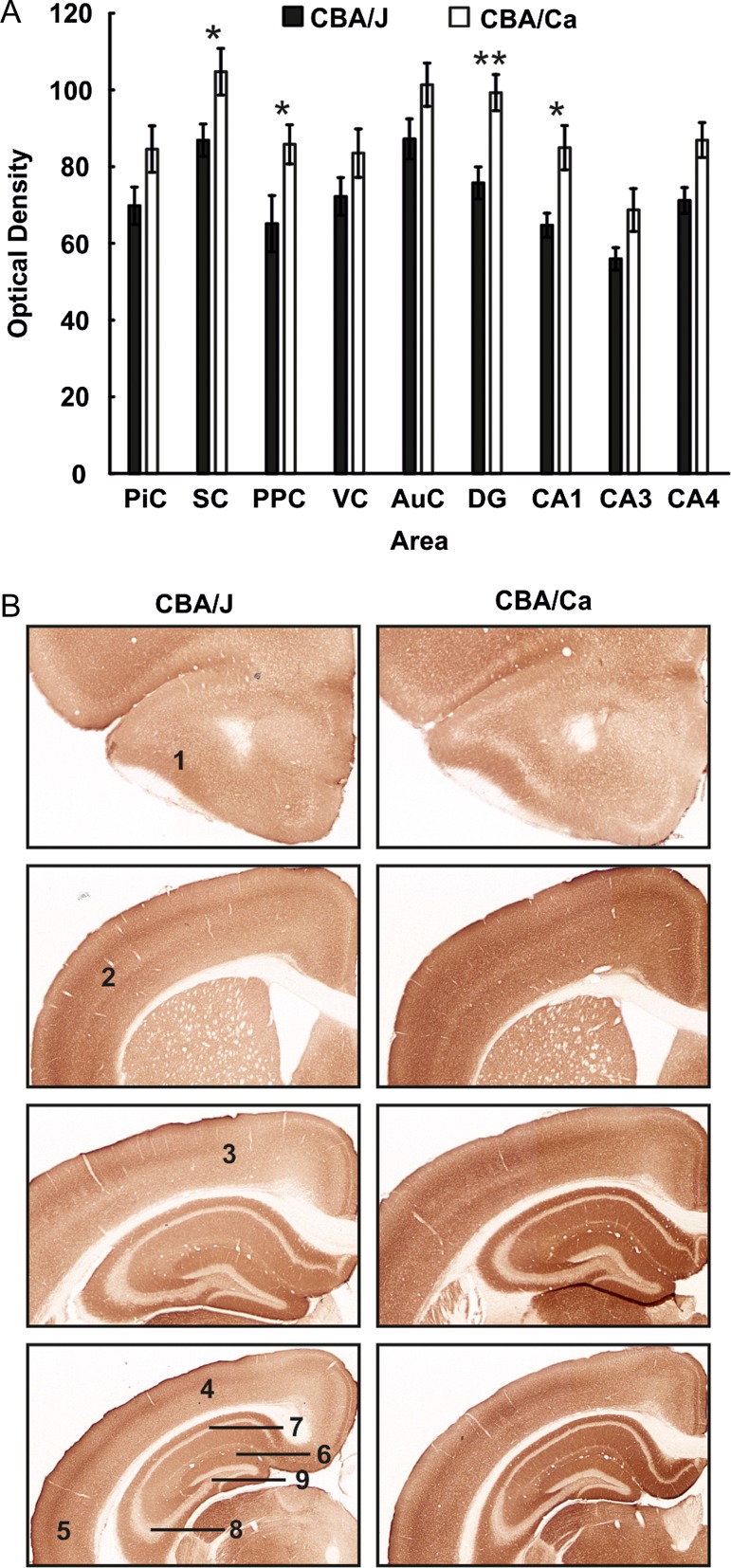
Changes in GABA-A expression are evident 4 months postnatally in CBAJ mice. (*A*) Bar charts represent mean GABA-A receptor optical densities in different brain regions 4 months postnatally in CBA/J mice compared with (CBA/CaOlaHsd) controls. Receptor density is significantly decreased in the somatosensory cortex, posterior parietal cortex, dentate gyrus, and CA1. Data are means ± SEM. *P < 0.05; **P < 0.01; ***P < 0.001. (*B*) Photomicrographs of DAB-stained coronary sections showing GABA-A expression in CBA/J and control mice 4 months postnatally. 1: piriform cortex, 2: somatosensory cortex, 3: posterior parietal cortex, 4: visual cortex, 5: auditory cortex, 6: dentate gyrus, 7: CA1, 8: CA3, 9: CA4.

In 4-month-old CBA/J mice (*N* = 6), GABA-A receptor density was decreased in the primary somatosensory cortex, the posterior parietal cortex, the dentate gyrus, and the CA1 region (Fig. 3) compared with CBA/CaOlaHsd mice (*N* = 6) (Table 1). GABA-B receptor expression showed no significant differences between CBA/J (*N* = 6) and CBA/CaOlaHsd mice (*N* = 6) (Supplementary Fig. 1, Supplementary Table 1), with the exception of the piriform cortex, where a significant increase was evident in 4-month-old CBA/J mice (Table 1).

### The Stimulus–Response Relationship of Hippocampal Field Potentials is Altered in the Hippocampus of 2-month-old, but not 4-month-old CBA/J Mice

**Figure 4.**
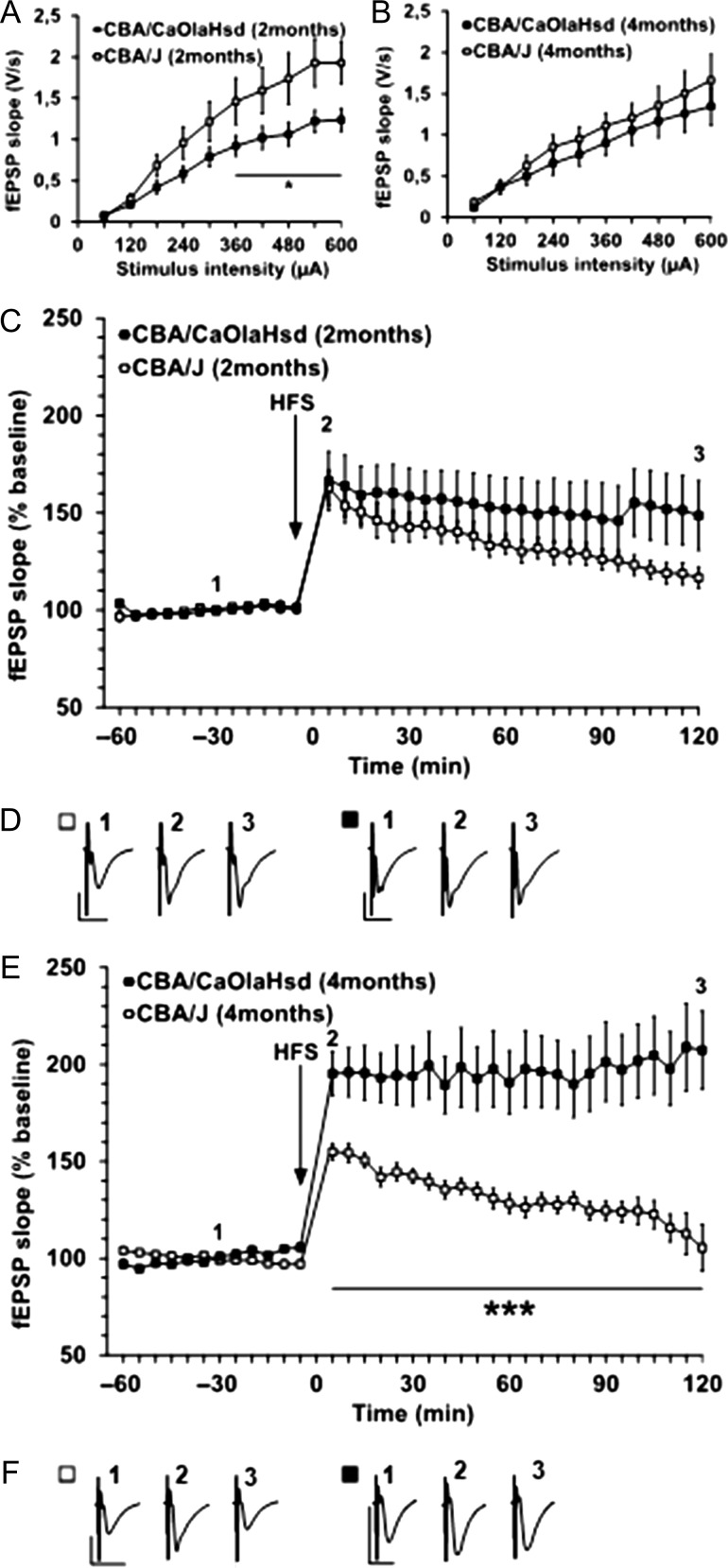
Synaptic plasticity is impaired in CBA/J mice. (*A*) Comparison of input–output responses in 2-month-old CBA/J and control mice revealed that fEPSP evoked in the stimulus intensity range of 360–600 μA were significantly higher in CBA/J mice. (*B*) Comparison of input–output responses in 4-month-old CBA/J and control mice found no significant differences in fEPSP evoked in the stimulus intensity range of 60–600 μA. (*C*) High-frequency stimulation (HFS) elicits LTP in 2-month-old CBA/J and control (CBA/CaOlaHsd) mice, which is not significantly different. (*D*) Analog examples show fEPSP recorded from 2-month-old CBA/J and control mice at the time points indicated in the graph shown in “A.” Vertical scale bar: 1 mV; horizontal scale bar: 10 ms. (*E*) HFS results in robust LTP in 4-month-old control mice. By contrast, CBA/J mice show a significantly impaired response to HFS: although potentiation results, both the induction and maintenance phases are significantly smaller in magnitude than that elicited in control mice. By 120-min post-HFS, evoked potentials returned to pre-HFS levels. (*F*) Analog examples show fEPSP recorded from 4-month-old CBA/J and control mice at the time points indicated in the graph shown in “A.” Vertical scale bar: 1 mV; horizontal scale bar: 10 ms.

Given that the hippocampus was the structure that showed the earliest change in receptor expression in 2-month-old CBA/J mice, and given that, by 4 postnatal months, this local increase in GluN2B subunit expression had spread to multiple hippocampal subregions and all sensory cortices along with more localized changes in GABA-A expression, we explored whether the hippocampus shows changes in its stimulus–response relationship at these postnatal time points. We recorded field potentials from the CA1 region of 2-month-old CBA/J mice (*N* = 6, *n* = 12, where “N” corresponds to animal number and “n” to slice number) and observed a higher sensitivity to stimulus intensity in the input–output curve, with effects becoming significant with intensities of 360 μA and higher compared with CBA/CaOlaHsd mice (*N* = 6, *n* = 12) (ANOVA: *F*(9 198) = 3.3318, *P* = 0.00082) (Fig. 4*A*). By 4 months postnatally, this difference in sensitivity was no longer present (ANOVA: *F*(9, 342) = 0.44550, *P* = 0.90964, *N* = 9, *n* = 19 for each cohort) (Fig. 4*B*).

### Hippocampal LTP is Profoundly Impaired 4 Months Postnatally in CBA/J Mice

We wondered whether the shift to the right of the input–output relationship in 2-month-old CBA/J mice might be associated with alterations in the ability of the hippocampus to express LTP: Intrinsic elevations of hippocampal excitability can lead to impairments in hippocampal LTP (Grüter et al. 2015). Thus, we assessed to what extent HFS of the Schaffer collaterals results in LTP in the CA1 region of 2-month-old CBA/J mice. Here, HFS resulted in LTP in CBA/J mice that was not statistically significant from LTP elicited in CBA/CaOlaHsd mice (ANOVA: *F*(23, 368) = 0.37498, *P* = 0.99669, *N* = 6, *n* = 9 for both cohorts) (Fig. 4*C*). The tendency toward a slightly weaker LTP magnitude in the time points recorded from 100 min after HFS onward was not significant (ANOVA: *F*(4, 64) = 0.02518, *P* = 0.99874). Thus, the change in stimulus–response sensitivity that occurred in the hippocampus of 2-month-old CBA/J mice did not affect the ability of the hippocampus to express LTP.

By contrast, by 4 months postnatally, CBA/J mice exhibited profoundly impaired LTP in comparison to LTP elicited in CBA/CaOlaHsd mice (ANOVA: *F*(23, 483) = 3.0280, *P* < 0.0001, *N* = 9, *n* = 10 for both cohorts). (Fig. 4*E*), even though at this postnatal age, no changes in the stimulus–response relationship were evident in the mice (Fig. 4*A*). Here, both the induction and the maintenance phases of LTP were affected (Fig. 4*E*). Statistical evaluation of LTP in 2- and 4-month-old CBA/CaOlaHsd mice revealed that there was no age-dependent change in LTP in the control mice (ANOVA: *F*(1, 20) = 2.4050, *P* = 0.13663, *N* = 6, *n* = 9).

### CBA/J Mice Display Impairments in Object Recognition Memory

To clarify whether CBA/J mice exhibit alterations in hippocampus-dependent memory, we tested them in an object recognition task, which has been shown in the past to recruit hippocampal involvement (Fortin et al. 2002; Manns and Eichenbaum 2006, 2009; Manns et al. 2007; Farovik et al. 2009; Goh and Manahan-Vaughan 2013).

**Figure 5.**
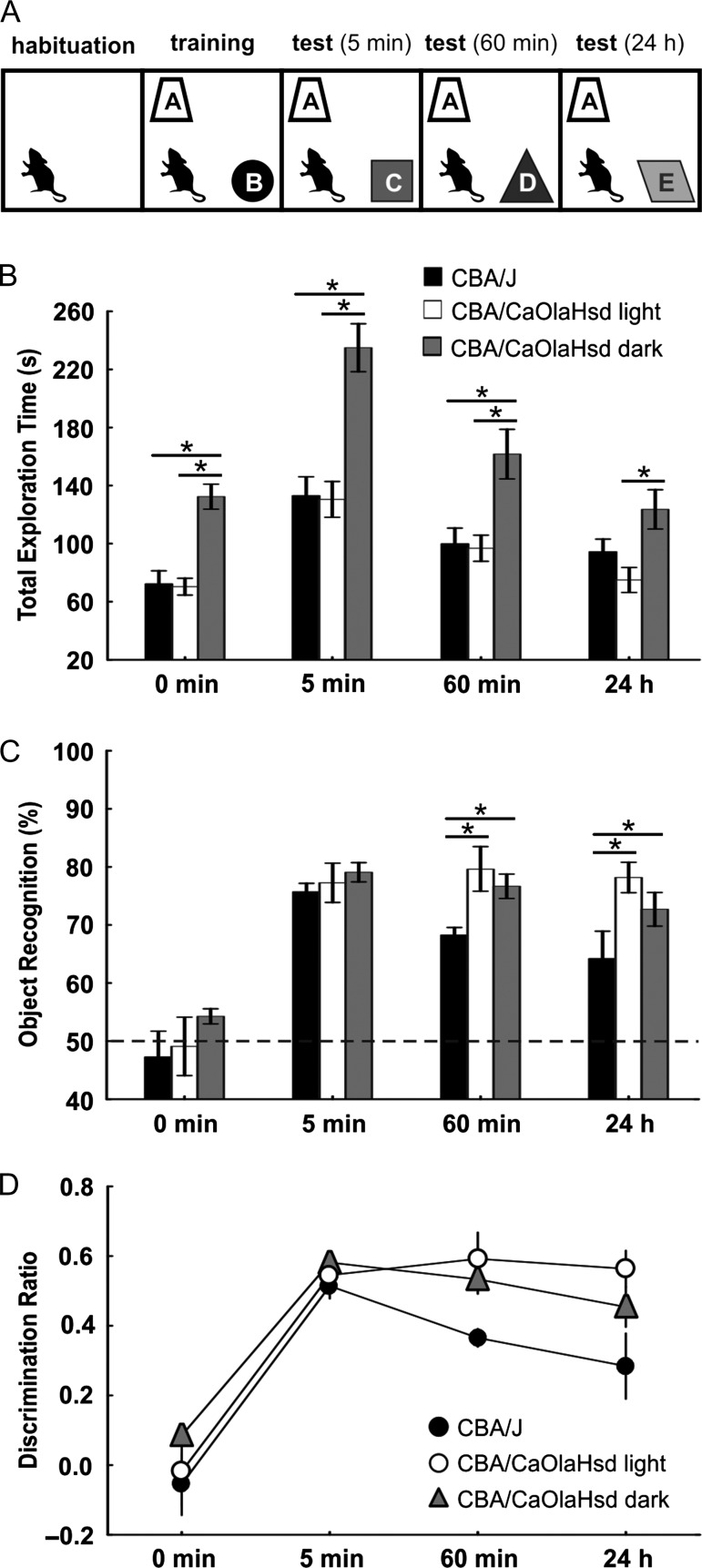
Novel object recognition memory is impaired in congenitally blind mice. (*A*) Schema of the object recognition task. Animals habituated to the environment on the day before training. During training, animals were exposed to 2 distinct objects (A and B). Five minutes, 60 min, and 24 h after this experience, exploration of object A was compared with exploration of a new object (C,D,E). (*B*) Bar charts show the total exploration time of 4–5-month-old CBA/J and control (CBA/CaOlaHsd) mice in a novel object recognition task. Behavior of the blind (CBA/J) mice was compared with that of CBA/CaOlaHsd mice under both illuminated and dark conditions. CBA/J and CBA/CaOlaHsd mice which explored under illuminated conditions engaged in equivalent levels of total exploration time during both the acquisition and test trials. Exploration times of CBA/CaOlaHsd mice in darkness was significantly different from both CBA/J mice and CBA/CaOlaHsd mice which explored under illuminated conditions. *P < 0.05. (*C*) Bar charts show the recognition indices of CBA/J and CBA/CaOlaHsd mice (under illuminated and dark conditions) during the object recognition task. The recognition index is expressed as a percentage of the total exploration time. The 50% line represents no preference for either object, values above the line represent a preference for the novel object. The recognition indices 5 min after initial exposure reveal that both strains retained a memory of the original object exposure and show a significant preference for the novel object. Although the CBA/J strain discriminates the new from the familiar object 60 min and 24 h after the first (training) exposure, object recognition is significantly poorer than that observed in the CBA/CaOlaHsd mice, both under illuminated and dark conditions. *P < 0.05. (*D*) The line graph shows the discrimination ratios of CBA/J and CBA/CaOlaHsd mice during the object recognition task. The discrimination ratio (DR) was calculated by dividing the difference of time spent at the novel object (*T*_N_) and the familiar object (*T*_F_) by the total exploration time: DR = (*T*_N_ − *T*_F_)/(*T*_N_ + *T*_F_). Values above 0 indicate a preference for the novel object, values under 0 a preference for the familiar object. No object preference was exhibited by the strains during the training phase (0 min). Five minutes after the training exposure, DRs were equivalent in CBA/J mice and CBA/CaOlaHsd mice (under illuminated and dark conditions). Both CBA/CaOlaHsd cohorts significantly outperform CBA/J mice, 60 min, and 24 h after the first (training) exposure to the objects.

Mice engaged in an object recognition task (Fig. 5*A*) and their exploration of a familiar and novel object was assessed 5 min, 60 min, and 24 h after original exposure to the subsequently familiar object. During training and all test conditions, the total time spent exploring the objects was equivalent in CBA/J mice (*n* = 8) and CBA/CaOlaHsd mice that explored under illuminated conditions (*n* = 8) (Fig. 5*B*). CBA/CaOlaHsd mice that explored in darkness exhibited significantly higher exploration times (*n* = 8, *P* < 0.05) (ANOVA for training phase: *F*_2,24_ = 19.862, *P* < 0.001, at 5-min test: *F*_2,25_ = 16.893, *P* < 0.001, at 60-min test: *F*_2,24_ = 6.603, *P* = 0.005, at 24-h test: *F*_2,24_ = 4.786, *P* = 0.018) (Fig. 5*B*). All animals exhibited recognition indices that reflected a memory of the old, and recognition of the novel object (Fig. 5*C*). Whereas the recognition indices of CBA/J mice were indistinguishable from CBA/CaOlaHsd mice 5 min after training, their indices were significantly poorer 60 min (each *P* < 0.05, *t*-test) and 24 h after training compared with the control mice (tested under both illuminated conditions and darkness) (each *P* < 0.05, *t*-test) (Fig. 5*C*). Examination of the object discrimination ratios also indicated that object recognition memory was impaired in CBA/J mice both 60 min and 24 h after initial object exposure (Fig. 5*D*). A multifactorial ANOVA with repeated measures confirmed the overall significance of results obtained 60 min (*F*_2,24_ = 5.086, *P* = 0.014) and 24 h (*F*_2,24_ = 3.731, *P* = 0.039) after training.

## Discussion

We explored the functional consequences of the early manifestation of blindness in CBA/J mice which congenitally express the rd1 (rd1) mutation of the *PDE6B *gene that promotes loss of rods through apoptosis (Lolley et al. 1977; Chang et al. 1993; Farber 1995). In particular, we explored to what extent NMDAR and GABA receptor expression changes in primary and associational sensory cortices, as well as in the hippocampus, and investigated if hippocampus-dependent learning and information encoding is altered. We compared CBA/J mice with CBA/CaOlaHsd mice that have no reported deficits in sensory or cognitive systems (Brooks et al. 2004, 2005; Walton et al. 2008; Jin and Ribelayga 2016; Heckman et al. 2017).

Scrutiny of the retina 4–5 weeks after birth revealed extensive degeneration in CBA/J mice that was accompanied by an absence of electroretinography (ERG) responses and thinning and cell loss within the retina. By contrast, CBA/CaOlaHsd mice exhibited a healthy fundus, a normal ERG response, and retinal cell layers and organization corresponding to healthy tissue. A major finding of this study is that by 2 postnatal months, reorganization of the hippocampus became apparent in the form of localized, increased hippocampal expression of the GluN2B subunit of the NMDAR that is accompanied by changes in the stimulus–response relationship of hippocampal field potentials. By 4 postnatal months, the increase in GluN2B expression had spread to all sensory and association cortices, as well as to all subregions of the hippocampus. Brain structure-specific elevations in GABA-B and localized decreases in GABA-A expression were also apparent. These changes in NMDAR and GABA receptor expression were accompanied by profound deficits in hippocampal LTP and hippocampus-dependent learning. These findings suggest that the receptor reorganization that is elicited by early vision loss, also has functional consequences for synaptic encoding properties of the hippocampus that may reflect a gradual adaptation of the hippocampus to the absence of the visual modality.

CBA/J mice exhibited deficits in object recognition memory that became evident 60 min after initial exposure to the objects. Exploration behavior in the test chamber was equivalent in CBA/J mice and CBA/CaOlaHsd mice that explored under illuminated conditions. Interestingly, exploration levels in the CBA/CaOlaHsd mice in darkness were higher than when they explored under illumination conditions, and also higher than those of CBA/J mice. This suggests that CBA/CaOlaHsd mice may have been less confident when they explored in darkness and thus spent more time investigating the objects. This had a slight, but non-significant negative effect on their memory of the objects (Fig. 5*C*). Nonetheless, their object memory remained significantly better than the CBA/J mice. Taken together this suggests that CBA/J mice developed a less accurate memory of the objects compared with control mice. The object recognition test conditions implemented in the current study are known to impact upon hippocampal information encoding and storage (Manns et al. 2007; Goh and Manahan-Vaughan 2013; Hoang et al. 2018), suggesting that these deficits may have been mediated by impaired hippocampal information processing.

By 2 months postnatally, CBA/J mice exhibited a decline in magnitude of the later phase of LTP that was significant compared with CBA/CaOlaHsd mice. Although the LTP we detected in the CBA/CaOlaHsd mice was quite pronounced, we do not believe that the LTP was near to saturated levels, particularly because the mice used in this study were not kept in an enriched environment that can indeed lead to substantially improved LTP (Novkovic, Mittmann et al. 2015; Novkovic, Heumann et al. 2015). At the time point of the behavioral study, they were completely naïve to behavioral experiences of the kind we tested. For this reason, it is also unlikely that cumulative effects on LTP (resulting from cumulative spatial learning) could have occurred (Manns et al. 2007; Straube et al. 2003). Nonetheless, the shift to the right in the I–O relationship that we detected in 2-month-old CBA/J mice might have affected the ability of the hippocampus to express LTP: global elevations of hippocampal excitability can be associated with deficits in LTP (Grüter et al. 2015). Despite this, no significant change in LTP was detected in 2-month-old mice that had shown this elevation of excitability. By contrast, in 4-month-old CBA/J mice, where LTP was substantially impaired, their input–output relationship was unchanged compared with CBA/CaOlaHsd mice. This suggests that the early changes in hippocampal excitability may reflect reorganizational changes due to the lack of visual input, and that LTP saturation or global elevations in excitability are not the instigators of the LTP deficits that we detected in CBA/J mice.

GluN2B may play a particular role in the support of cortical reorganization and adaptation in blindness. This NMDAR subunit plays an important role in many forms of hippocampal synaptic plasticity (Köhr et al. 2003; Liu et al. 2004; Massey et al. 2004; Ballesteros et al. 2016) and also contributes to synaptic plasticity in the visual cortex (Sawtell et al. 1999; Cho et al. 2009; Sidorov et al. 2015). Visual plasticity during the critical period (Lee et al. 2015) particularly involves the GluN2B subunit. Although GluN2B-dependent plasticity has been reported in the adult visual cortex under circumstances where Mg^2+^ treatment was used to manipulate GluN2B subunit expression (Liu et al. 2015), it has been shown that a systematic increase of GluN2A subunits and a decline of GluN2B subunits accompanies the transition from early postnatal stages through adulthood (Carmignoto and Vicini 1992). This has been proposed to account for the age-dependent decline in adult visual cortical plasticity. Furthermore, visual experience preferentially promotes the expression of GluN2A-containing NMDAR in the visual cortex (Quinlan et al. 1999; He et al. 2006), suggesting that the GluN2A subunit may underlie adult visual plasticity in healthy animals.

Short-term consequences of visual deprivation comprise an upregulation of excitatory synapses and enhanced LTP in the primary visual cortex (Kirkwood et al. 1996; Goel and Lee 2007). In juvenile mice, brief visual deprivation also causes transient increases in GluN2B and prolonged deprivation causes decreases in GluN2A expression (Chen and Bear 2007). The thresholds for LTP induction are lower for GluN2A-containing NMDAR (Berberich et al. 2007; Ballesteros et al. 2016), which could explain why LTP is enhanced in juvenile mice that have been visually deprived for a few weeks. Our results suggest that “prolonged” visual deprivation triggers a different kind of NMDAR reorganization that primarily depends on the upregulation of GluN2B expression. Effects appear to begin in the hippocampus, and specifically in the CA4 region where elevations in GluN2B expression become evident in 2-month-old CBA/J mice.

Although we did not scrutinize synaptic plasticity in the visual cortex in our study, we observed that 4-month-old CBA/J mice, which become completely blind by 4 weeks postnatally, exhibited a loss of “hippocampal” LTP. Studies that examined the consequences of early visual deprivation, have, to our knowledge, not examined possible changes in the hippocampus. Thus, our finding of impaired LTP is a completely novel observation. It is quite possible that the strongly impaired LTP was caused by the increases in GluN2B expression that occurred in the hippocampus by 4 months postnatally. This interpretation is based on the fact that at 2 months, only the later phase of LTP was impaired, at which time GluN2B receptor levels were unchanged in the CA1 region, where LTP was induced. Furthermore, GluN2B-containing NMDARs change the thresholds for induction of LTP: here, a more intense stimulation pattern is required to induce LTP than that needed to activate GluN2A-containing NMDAR (Berberich et al. 2007; Ballesteros et al. 2016).

Prolonged visual deprivation resulted in GluN2B upregulation, not only just in the visual cortex and hippocampus but also in all of the primary and secondary sensory cortices assessed in our study. This may correspond to cortical reorganization that is known to occur following visual deprivation in humans (Bavelier and Neville 2002; Burton 2003; Frasnelli et al. 2011) and suggest that the NMDAR is a key component underlying this kind of reorganization. The question arises, however, as to why GluN2B and not GluN2A expression is upregulated under these conditions. Studies in the cat visual cortex have demonstrated that cortical reorganization that is initiated by retinal damage, triggers a return of the visual cortex to the early postnatal state (Takesian and Hensch 2013). This phase of postnatal development is associated with high relative levels of GluN2B in the visual cortex (Sheng et al. 1994). Furthermore, GluN2B is involved in the enablement of metaplasticity in the mouse visual cortex (Chen and Bear 2007). Thus, the increase in GluN2B expression may reflect initial crossmodal cortical adaptations to the absence of visual input. Although the long-term consequences of blindness in rodents have not been studied in this regard, studies in young (3–4-week-old) mice have shown that brief (ca. 1 week) visual deprivation enhances frequency sensitivity in the auditory cortex (Petrus et al. 2014). Others have reported that an increase in excitability in the visual cortex is accompanied by a decrease in the somatosensory cortex (Goel et al. 2006). We feel our results are not directly comparable to findings in young mice following brief visual deprivation, however: blindness emerged in our mice at 4 weeks postnatally and this corresponds to the time point at which transient visual deprivation is typically conducted. By 2 months postnatally, where visual deprivation lasting 4 weeks can be expected to have occurred, we saw no receptor adaptation (bar a highly localized upregulation of GluN2B in the CA4 region) at 2 months postnatally. This suggests that the rapid adaptation effects reported following transient visual deprivation of juvenile mice are shortlived.

By 4 months postnatally, CBA/J mice not only exhibited widespread upregulation of GluN2B but also showed lower levels of GABA-A in the somatosensory cortex, piriform cortex, as well as in the hippocampal dentate gyrus and CA1 regions. Reduced GABA-A receptor expression can be expected to result in less effective inhibitory transmission that could result in increased excitability. Curiously, however, we saw increased stimulus sensitivity in the hippocampus at 2 months and not 4 months of age. The changes in input–output responses at 2 months cannot be explained by changes in the expression of other plasticity-related receptors: we observed no expression changes in metabotropic glutamate receptors, GluN1, or GluN2A. GABA-B receptor expression was also unchanged, with the exception of increased expression in the piriform cortex at 4 months. We did not examine for changes in the AMPA receptor, however. This receptor has been reported to underlie changes in auditory cortex sensitivity to transient visual deprivation of rodents (Goel et al. 2006). The same authors reported, by contrast, that visual deprivation has no effect on AMPAR expression in the frontal cortex, suggesting that cortical effects are disparate (Goel et al. 2006).

Although decreased immunoreactivity of GABA positive neurons has been reported in the primary visual cortex of dark reared mice (Benevento et al. 1995) and in cats with retinal lesions (Rosier et al. 1995), the reduction in GABA-A expression that we observed in 4-month-old blind mice did not affect the visual cortex, rather it was evident in the somatosenosry cortex and the posterior parietal cortex. GABAergic interneurons modulate cortical excitation following visual deprivation, suggesting a specific role for crossmodal plasticity (Desgent and Ptito 2012). Our findings suggest that crossmodal adaptation, related to increased perceptual acuity, was already underway at 4 postnatal months. However, the impairment of both hippocampal synaptic plasticity and hippocampus-dependent memory suggest that adaptation to blindness may not yet be complete at this stage of development. Rather, our data suggest that after 3 months of blindness, the hippocampus has not yet learned to cope with the loss of the visual modality. This is an important finding, given that it is well established that blind individuals develop an elevated sense of tactile (Van Boven et al. 2000; Goldreich and Kanics 2003), olfactory (Cuevas et al. 2009), and auditory acuity (Röder et al. 1999) that allows them to acquire a very precise representation of space and spatial experience (Fortin et al. 2008).

The precision of spatial acuity of blind humans depends on the time point in life at which adaptive training began, which younger individuals (<12 years old) having better prospects than older individuals (Fiehler et al. 2009). By contrast, it has been reported that individuals that become blind early in life are poorer in judging distances between landmarks in a spatial navigation task (Rieser et al. 1992). The mice we studied here developed congenital blindness rapidly after birth. Although we detected an advanced state of retinal degeneration at 4–5 postnatal weeks, we cannot exclude that the brief visual input that the mice may have received in the interval between eye opening (10–14 days postnatally) and retinal degeneration may have impacted on their ability to adapt to visual loss. The possibility remains that very long-term improvements in behavioral learning and hippocampal synaptic plasticity may develop as the animals advance into mature and late adulthood.

In conclusion, prolonged visual deprivation that is associated with the onset of complete blindness in juvenile mice is associated with a large-scale upregulation of GluN2B expression through primary and secondary sensory cortices and the hippocampus, as well as a downregulation of GABA-A receptor expression that is localized to the somatosensory and posterior parietal cortices, and hippocampal dentate gyrus and CA1 regions. All other plasticity-related proteins studied (GluN1, GluN2A, GABA-B, mGlu1, mGlu2.3, mGlu5) were unaffected, except for a local GABA-B expression increase in the piriform cortex at 4 months. These changes in receptor expression are accompanied by a significant loss of hippocampal LTP and of hippocampus-dependent learning. Taken together these findings indicate that visual cortical and crossmodal adaptation to prolonged visual deprivation is a gradual process that initially results in impaired hippocampal information encoding and spatial learning.

## Supplementary Material

Supplementary Data
