# Supplementary material for: Early Loss of Vision Results in Extensive Reorganization of Plasticity-Related Receptors and Alterations in Hippocampal Function That Extend Through Adulthood
Source: Cereb Cortex. 2018 Dec 7;29(2):892–905. doi: 10.1093/cercor/bhy297 (PMC6319173; doi:10.1093/cercor/bhy297)
Supplement: Supplementary Data [file bhy297supplement_1.zip › bhy297_Feldmann_supplementary_legends.docx]

**Supplementary Figure and Table Legends**

**Supplementary Table 1. Mean optical densities of cortical neurotransmitter receptors in CBA/J and CBA/CaOlaHsd**  **mice 2 months and 4 months postnatally**

**Supplementary Table 2. Summary of the numbers of animals used for the different experimental approaches.**

**N**: number of animals used in all experimental approaches, **n**: number of slices used in *in vitro* electrophysiological experiments.

**Supplementary Figure 1. GluN1, GluN2A and GluN2B expression in CBA/J and control mice 2 and 4 months postnatally.**

**A-B.** GluN1 2 and 4 months postnatally. No significant differences were observed.

**C-D.** GluN2A 2 and 4 months postnatally. No significant differences were observed.

**E.** GluN2B 2 months postnatally. Receptor density was significantly increased in CA4 in CBA/J mice compared to control (CBA/CaOlaHsd) mice.

**F.** GluN2B 4 months postnatally. Receptor density was significantly increased across all hippocampal and cortical areas of interest in CBA/J mice compared to control mice.

Bar charts represent mean receptor optical density ± SEM. *p < 0.05; **p < 0.01; ***p < 0.001

**Supplementary Figure 2. GABA-A and GABA-B receptor expression in CBA/J and control mice 2 and 4 months postnatally.**

**A.** Optical density assessment of GABA-A receptors revealed that expression is equivalent 2 months postnatally in CBA/J and CBA/CaOlaHsd mice.

**B.** GABA-A expression 4 months postnatally is significantly decreased in CBA/J mice in the somatosensory cortex (SC), posterior parietal cortex (PPC), dentate gyrus (DG) and CA1.

**C-D.** Optical density assessment of GABA-B receptor expression, 2 and 4 months postnatally revelaed no difference at 2 months of age. At 4 months postnatally, receptor expression was equivalent in all regions studied, with the exception of the piriform cortex (PiC), where CBA/J mice exhibited elevated GABA-B receptor expression.

Bar charts represent mean receptor optical density ± SEM. *p < 0.05; **p < 0.01; ***p < 0.001

**Supplementary Figure 3. High resolution examples of GABA and NMDA receptor expression in the hippocampal CA1 region.**

**A.** – **E.** Immunohistochemical examples show receptor/ subunit expression corresponding to GluN1 (**A**), GluN2A (**B**), GluN2B (**C**), GABA-A(**D**), und GABA-B(**E**), in the hippocampal CA1 region using 40-fold magnification

**F.** Overview of the hippocampal section (using 5-fold magnification) from which the abovementioned GluN1 example was taken. The rectangle indicates the area of the CA1 region that was selected for 40-fold magnification (**A**).
