# Supplementary material for: Early Loss of Vision Results in Extensive Reorganization of Plasticity-Related Receptors and Alterations in Hippocampal Function That Extend Through Adulthood
Source: Cereb Cortex. 2018 Dec 7;29(2):892–905. doi: 10.1093/cercor/bhy297 (PMC6319173; doi:10.1093/cercor/bhy297)
Supplement: Supplementary Data [file bhy297supplement_1.zip › bhy297_Supplementary_Table_1.docx]

**Supplementary Table 1. Mean optical densities of cortical neurotransmitter receptors in CBA/J and control mice 2 months and 4 months postnatally**

|  | **Pic** | **SC** | | **PPC** | | **VC** | | **AuC** | | **DG** | | **CA1** | | **CA3** | | | **CA4** | |
| --- | --- | --- | --- | --- | --- | --- | --- | --- | --- | --- | --- | --- | --- | --- | --- | --- | --- | --- |
|  |  |  | |  | |  | |  | |  | |  | |  | | |  | |
| **2 months** |  |  | |  | |  | |  | |  | |  | |  | | |  | |
|  |  | |  | |  | |  | |  | |  | |  | |  | |  | |
| **GluN1** |  | |  | |  | |  | |  | |  | |  | |  | |  | |
| CBA/J | 13.72 | | 13.17 | | 14.17 | | 12.93 | | 13.08 | | 9.88 | | 12.07 | | 17.34 | | 12.70 | |
| CBA/CaOlaHsd | 13.44 | | 15.20 | | 15.51 | | 13.79 | | 14.83 | | 11.83 | | 13.89 | | 19.65 | | 13.45 | |
| p | 0.963 | | 0.762 | | 0.827 | | 0.896 | | 0.795 | | 0.720 | | 0.788 | | 0.672 | | 0.907 | |
| **GluN2A** |  | |  | |  | |  | |  | |  | |  | |  | |  | |
| CBA/J | 16.28 | | 18.08 | | 18.28 | | 15.94 | | 17.45 | | 13.57 | | 15.16 | | 20.20 | | 15.59 | |
| CBA/CaOlaHsd | 17.28 | | 17.05 | | 14.60 | | 13.66 | | 13.84 | | 14.03 | | 14.45 | | 18.95 | | 14.29 | |
| p | 0.561 | | 0.566 | | 0.055 | | 0.242 | | 0.064 | | 0.802 | | 0.682 | | 0.439 | | 0.482 | |
| **GluN2B** |  | |  | |  | |  | |  | |  | |  | |  | |  | |
| CBA/J | 23.01 | | 32.98 | | 39.23 | | 38.68 | | 33.85 | | 24.04 | | 36.51 | | 32.68 | | 28.85 | |
| CBA/CaOlaHsd | 23.92 | | 26.59 | | 34.40 | | 31.19 | | 28.41 | | 17.1 | | 31.11 | | 28.06 | | 18.28 | |
| p | 0.820 | | 0.183 | | 0.280 | | 0.111 | | 0.254 | | 0.127 | | 0.258 | | 0.326 | | 0.022* | |
| **GABA-A** |  | |  | |  | |  | |  | |  | |  | |  | |  | |
| CBA/J | 47.19 | | 68.35 | | 56.85 | | 60.67 | | 67.20 | | 56.39 | | 48.74 | | 41.87 | | 49.42 | |
| CBA/CaOlaHsd | 47.56 | | 66.58 | | 62.92 | | 67.27 | | 67.15 | | 57.08 | | 51.71 | | 37.79 | | 48.99 | |
| p | 0.960 | | 0.838 | | 0.468 | | 0.452 | | 0.995 | | 0.931 | | 0.723 | | 0.585 | | 0.955 | |
| **GABA-B** |  | |  | |  | |  | |  | |  | |  | |  |  | |  |
| CBA/J | 22.14 | | 21.95 | | 20.1 | | 22.90 | | 24.55 | | 27.28 | | 23.42 | | 28.19 | 27.87 | |  |
| CBA/CaOlaHsd | 16.27 | | 19.17 | | 25.05 | | 23.97 | | 20.42 | | 23.31 | | 20.72 | | 21.79 | 25.80 | |  |
| p | 0.131 | | 0.470 | | 0.223 | | 0.772 | | 0.304 | | 0.307 | | 0.491 | | 0.113 | 0.557 | |  |
|  |  | |  | |  | |  | |  | |  | |  | |  |  | |  |
| **4 months** |  | |  | |  | |  | |  | |  | |  | |  | |  | |
|  |  | |  | |  | |  | |  | |  | |  | |  | |  | |
| **GluN1** |  | |  | |  | |  | |  | |  | |  | |  | |  | |
| CBA/J | 24.88 | | 23.68 | | 24.72 | | 22.20 | | 22.48 | | 22.39 | | 30.76 | | 35.20 | | 20.83 | |
| CBA/CaOlaHsd | 28.63 | | 26.10 | | 30.01 | | 18.20 | | 19.90 | | 21.31 | | 25.07 | | 27.01 | | 19.01 | |
| p | 0.54 | | 0.691 | | 0.399 | | 0.518 | | 0.678 | | 0.848 | | 0.356 | | 0.170 | | 0.748 | |
| **GluN2A** |  | |  | |  | |  | |  | |  | |  | |  | |  | |
| CBA/J | 19.33 | | 19.04 | | 18.26 | | 16.46 | | 16.49 | | 12.46 | | 14.75 | | 20.22 | | 12.62 | |
| CBA/CaOlaHsd | 16.75 | | 17.49 | | 18.66 | | 15.64 | | 14.84 | | 12.67 | | 13.80 | | 18.55 | | 12.39 | |
| p | 0.274 | | 0.499 | | 0.852 | | 0.682 | | 0.456 | | 0.9220.631 | | 0.631 | | 0.462 | | 0.913 | |
| **GluN2B** |  | |  | |  | |  | |  | |  | |  | |  | |  | |
| CBA/J | 30.12 | | 39.38 | | 46.93 | | 39.51 | | 35.38 | | 23.68 | | 37.72 | | 36.61 | | 22.93 | |
| CBA/CaOlaHsd | 12.27 | | 20.08 | | 26.93 | | 23.86 | | 20.03 | | 11.97 | | 23.28 | | 19.81 | | 10.25 | |
| p | <0.001* | | <0.001* | | <0.001* | | <0.001* | | <0.001* | | 0.003* | | <0.001* | | <0.001** | | 0.001* | |
| **GABA-A** |  | |  | |  | |  | |  | |  | |  | |  | |  | |
| CBA/J | 69.83 | | 86.89 | | 65.16 | | 72.23 | | 87.21 | | 75.75 | | 64.73 | | 55.97 | | 71.18 | |
| CBA/CaOlaHsd | 84.56 | | 104.76 | | 85.80 | | 83.51 | | 101.36 | | 99.29 | | 84.96 | | 68.71 | | 86.92 | |
| p | 0.074 | | 0.028* | | 0.014* | | 0.141 | | 0.063 | | 0.004* | | 0.016* | | 0.108 | | 0.067 | |
| **GABA-B** |  | |  | |  | |  | |  | |  | |  | |  |  | |  |
| CBA/J | 32.67 | | 28.81 | | 25.49 | | 28.09 | | 35.14 | | 36.46 | | 27.39 | | 39.04 | 43.69 | |  |
| CBA/CaOlaHsd | 22.72 | | 29.09 | | 24.57 | | 22.03 | | 30.93 | | 44.06 | | 31.27 | | 43.85 | 49.87 | |  |
| p | 0.020* | | 0.938 | | 0.801 | | 0.151 | | 0.300 | | 0.062 | | 0.364 | | 0.215 | 0.124 | |  |
