# Supplementary material for: Early Loss of Vision Results in Extensive Reorganization of Plasticity-Related Receptors and Alterations in Hippocampal Function That Extend Through Adulthood
Source: Cereb Cortex. 2018 Dec 7;29(2):892–905. doi: 10.1093/cercor/bhy297 (PMC6319173; doi:10.1093/cercor/bhy297)
Supplement: Supplementary Data [file bhy297supplement_1.zip › bhy297_Supplementary_Table_2_animals.docx]

| **Experimental approach** | **strain** | **age** | **N** | **n** |
| --- | --- | --- | --- | --- |
| retinal assessment | CBA/J | 4-5 weeks | 8 |  |
|  |  | 7-8 weeks | 8 |  |
|  | CBA/CaOlaHsd | 4-5 weeks | 8 |  |
|  |  | 7-8 weeks | 5 |  |
| Retinogram | CBA/J | 4-5 weeks | 4 |  |
|  | CBA/CaOlaHsd | 4-5 weeks | 4 |  |
| Immunohistochemistry | CBA/J | 2 months | 6 |  |
|  |  | 4 months | 5 – 6 |  |
|  | CBA/CaOlaHsd | 2 months | 6 |  |
|  |  | 4 months | 5 – 6 |  |
| electrophysiological response properties | CBA/J | 2 months | 6 | 12 |
|  |  | 4 months | 9 | 19 |
|  | CBA/CaOlaHsd | 2 months | 6 | 12 |
|  |  | 4 months | 9 | 19 |
| hippocampal LTP | CBA/J | 2 months | 6 | 9 |
|  |  | 4 months | 9 | 10 |
|  | CBA/CaOlaHsd | 2 months | 6 | 9 |
|  |  | 4 months | 9 | 10 |
| object recognition task | CBA/J | 16-20 weeks | 8 |  |
|  | CBA/CaOlaHsd  (illuminated, or dark conditions) | 16-20 weeks | 8 |  |
