# Supplementary figures and images for: Early Loss of Vision Results in Extensive Reorganization of Plasticity-Related Receptors and Alterations in Hippocampal Function That Extend Through Adulthood
Source: Cereb Cortex. 2018 Dec 7;29(2):892–905. doi: 10.1093/cercor/bhy297 (PMC6319173; doi:10.1093/cercor/bhy297)

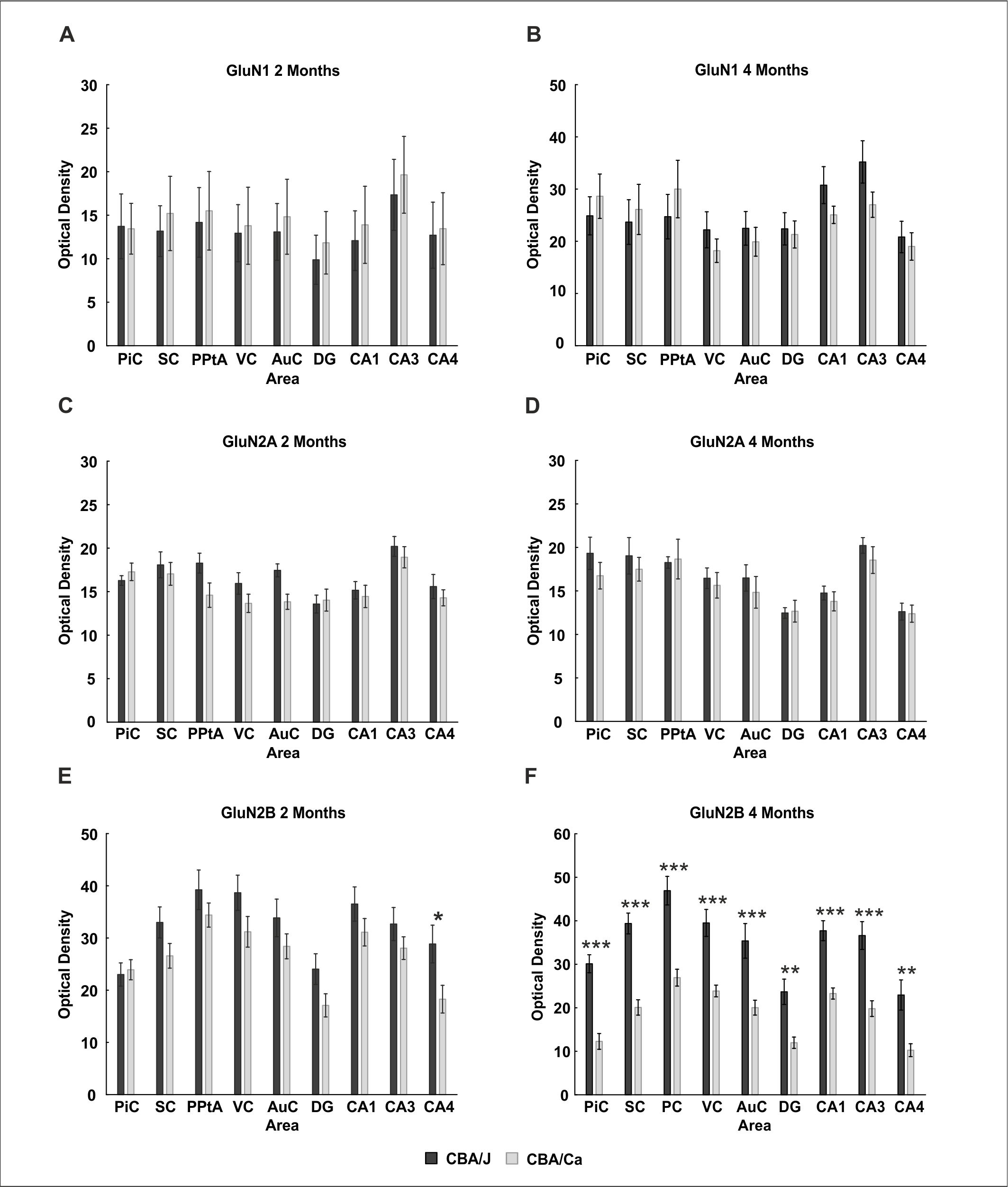

Supplement: Supplementary Data [file bhy297supplement_1.zip › bhy297_Supplementary_Figure_1_NMDAR.tif]

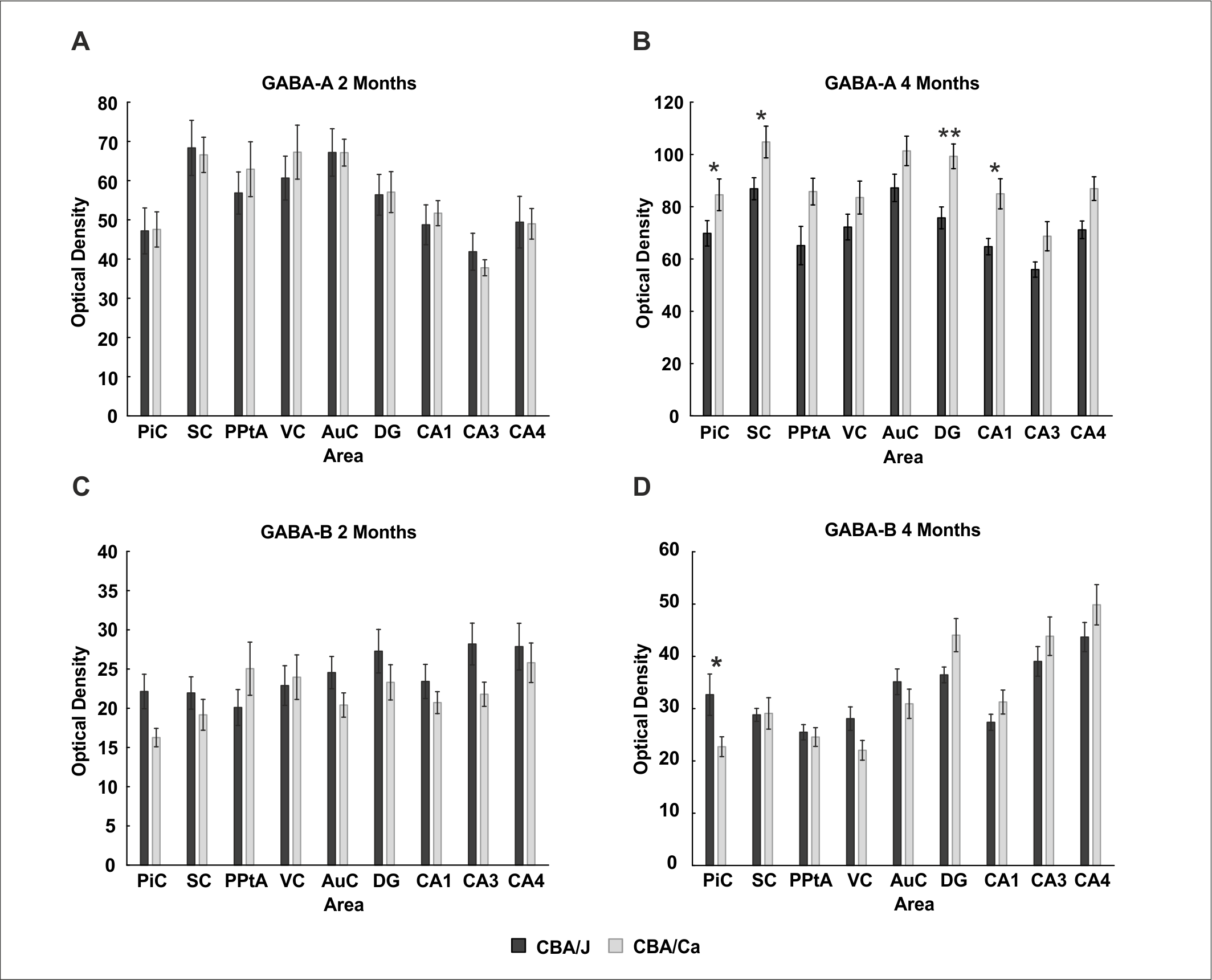

Supplement: Supplementary Data [file bhy297supplement_1.zip › bhy297_Supplementary_Figure_2_GABA.tif]

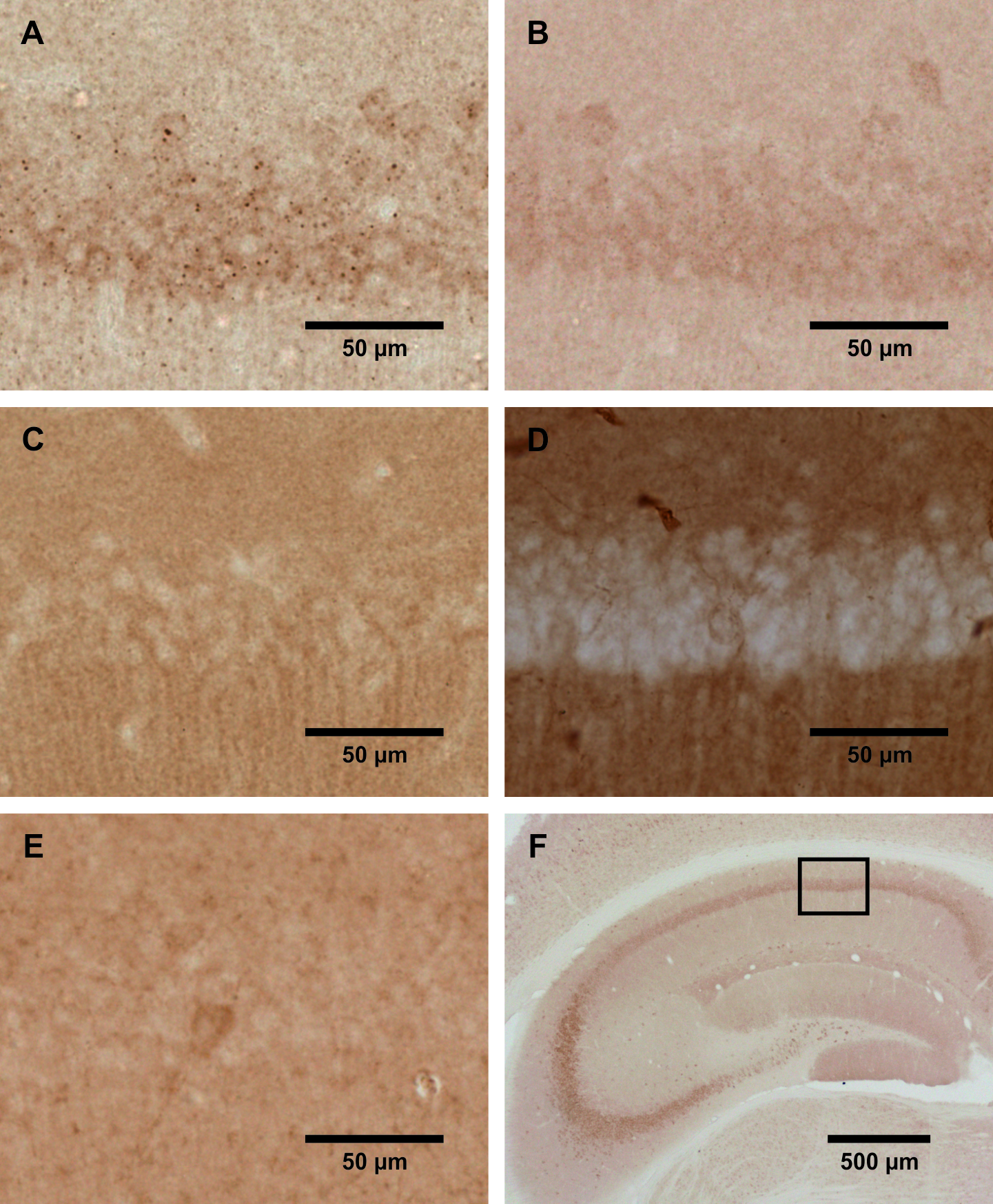

Supplement: Supplementary Data [file bhy297supplement_1.zip › bhy297_Supplementary_Figure_3_CA1_high_magnification.tif]
